# Efficacy of a moisturizer for pruritus accompanied by xerosis in patients undergoing dialysis: A multicenter, open‐label, randomized verification study

**DOI:** 10.1111/1346-8138.15950

**Published:** 2021-05-26

**Authors:** Yukie Yoshida, Akio Hirama, Kazumasa Hashimoto, Takeshi Sato, Noritsugu Yokota, Hidehisa Saeki, Momoyo Kishida, Hiroshi Nakamura, Akira Kanakubo, Shuichi Tsuruoka

**Affiliations:** ^1^ Kidney Disease Clinic of Nippon Medical School Bunkyo‐ku Japan; ^2^ Department of Nephrology Koyama Memorial Hospital Kashima Japan; ^3^ Department of Internal Medicine Moka Hospital Moka Japan; ^4^ Department of Dermatology Nippon Medical School Japan; ^5^ Medical Affairs Department Maruho Co., Ltd. Osaka Japan; ^6^ Department of Nephrology Nippon Medical School Bunkyo‐ku Japan

**Keywords:** chronic kidney disease, dialysis, heparinoid, pruritus, xerosis

## Abstract

Xerosis and pruritus are common in patients undergoing dialysis. These symptoms are treated with moisturizers, but limited evidence supports the efficacy of such treatment. Our exploratory study suggested the effectiveness of a heparinoid‐containing product for xerosis in dialysis patients. We conducted a multicenter, open‐label, randomized, before‐after, parallel‐group comparative study to verify the exploratory study results (Clinical Trial Registry: UMIN000029360). Seventy‐one Japanese patients undergoing dialysis with chronic kidney disease and xerosis were randomly assigned to receive a heparinoid‐containing product for 2 weeks (group A [*n* = 36]) or 8 weeks (group B [*n* = 35]). Patients were instructed to apply the study product based on the fingertip unit method. The efficacy endpoints were the water content of the stratum corneum (WCSC), skin dryness score, pruritus visual analog scale score, and Dermatology Life Quality Index. Safety was assessed by monitoring adverse events. The mean WCSC (arbitrary units) was 26.0 ± 9.6 in group A and 25.2 ± 10.0 in group B at the start of treatment (week 0), significantly increased to 39.0±12.5 in group A and 38.5 ± 11.0 in group B (*P* < 0.0001 for both vs week 0) by week 2, and then decreased only in group A. Thus, the WCSC at week 4 (the primary endpoint) remained significantly higher in group B (36.4 ± 12.2 vs 28.8 ± 10.4; *P* = 0.0068). Other endpoints improved during treatment with the study product. One patient developed a rash and erythema as treatment‐related adverse events. In conclusion, 8 weeks’ application of a heparinoid‐containing product was effective for xerosis in patients undergoing dialysis.

## 1 INTRODUCTION

Dry skin is observed in 50%–90% of patients with end‐stage renal disease and persists or even worsens despite dialysis.^1^ Pruritus is also reported in 12%–90% of patients with end‐stage renal disease.^1^ Suggested causes of pruritus in patients with end‐stage renal disease undergoing dialysis include dry skin, the influence of dialysis membranes, and the effect of increased circulating pruritic cytokines.^2^, ^3^ A descriptive study of the risk factors for pruritus in patients undergoing dialysis revealed more intense pruritus in patients with dry skin than in those with normal skin.^4^ Additionally, a greater number of dermatoses and longer duration of dialysis are reportedly associated with poorer scores on the Dermatology Life Quality Index (DLQI),^5^ a tool used to measure the quality of life (QOL) related to skin disease.^6^ For pruritus in particular, greater severity is reportedly associated with poorer QOL^7^ and higher mortality.^8^

Dry skin in patients undergoing dialysis is regarded as a type of xerosis,^9^ for which treatment with a moisturizer is recommended. In Japan, heparinoid‐containing products have been approved as prescription moisturizers and are used to treat xerosis associated with skin diseases such as atopic dermatitis, senile xeroderma, and dry skin in patients undergoing dialysis. Pruritus is typically treated with antihistamines,^10^ but there is reportedly no correlation between the severity of pruritus and blood histamine levels in patients undergoing dialysis.^11^ In fact, antihistamines are ineffective in many cases of pruritus. Specialized medicines for the treatment of pruritus in dialysis patients, such as kappa opioid receptor agonists, have recently also been used, but the symptoms are not completely suppressed. Given that moisturizers have been reported to be effective in patients with pruritus according to the 2020 clinical practice guidelines for the management of pruritus^12^ in Japan, and that xerosis may cause pruritus in patients undergoing dialysis, the application of moisturizers has been found to be effective for both xerosis and pruritus. However, evidence to support this effectiveness is limited to the results of a few observational or experimental studies, therefore stronger evidence on this topic is needed.

We previously conducted an exploratory study of 12 patients with chronic kidney disease and xerosis undergoing hemodialysis, and the results suggested the effectiveness of a moisturizer for xerosis and associated pruritus in these patients.^13^ In that study, we evaluated 2‐week (group A) versus 4‐week (group B) application of a heparinoid‐containing product with weekly measurement of the water content of the stratum corneum (WCSC). The WCSC increased in both groups up to week 2 and was then maintained in group B up to week 4, but decreased in group A after week 2. However, a significant difference in the WCSC at week 4 (the primary endpoint of the study) was not observed between the two groups, possibly because of an insufficient sample size. Pruritus also improved during treatment with the study product and deteriorated after the end of the moisturizer application in group A, with a significant difference between the two groups at week 4 (*P* = 0.01, paired *t*‐test).

The present confirmatory study was designed with an adequate sample size based on the preceding study results. The DLQI was used as an additional endpoint to investigate the influence of a heparinoid‐containing product on QOL.

## 2 METHODS

### 2.1 Ethical considerations

This study was approved by the ethics review committees of Nippon Medical School (16 June 2017; Approval No. 229006) and Adachi Kyosai Hospital (24 August 2017), and was registered with the University Hospital Medical Information Network (UMIN) Clinical Trials Registry (No. UMIN000029360). The study was conducted in compliance with the Ethical Guidelines for Medical and Health Research Involving Human Subjects and the ethical principles of the Declaration of Helsinki.

### 2.2 Institutions and study period

This study was conducted at the Kidney Disease Clinic of Nippon Medical School, Koyama Memorial Hospital, and Moka Hospital from October 2018 to May 2019.

### 2.3 Study design

**FIGURE 1:**
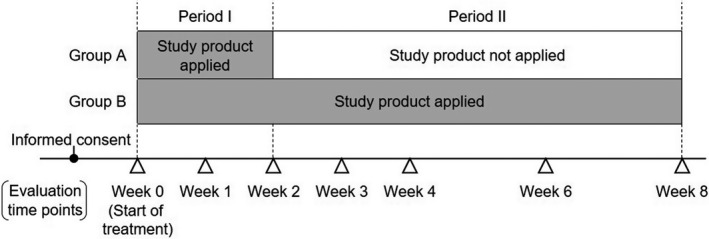
Study schedule. A heparinoid‐containing product was applied for 2 weeks in group A and 8 weeks in group B. Informed consent was obtained before the start of treatment. Efficacy assessments were performed at weeks 0, 1, 2, 3, 4, 6, and 8. In patients who discontinued the treatment, final assessments were performed on the day of discontinuation

This was a multicenter, open‐label, randomized, before–after, parallel‐group comparative study. The study schedule is shown in Figure 1. Participants were randomized in a 1:1 ratio to groups A and B. In group A, the study product was applied for 2 weeks (period I), while in group B, the study product was applied for 8 weeks (2 weeks in period I plus 6 weeks in period II). Randomization of the participants was conducted before initiation of treatment. Study assessments were performed at weeks 0, 1, 2, 3, 4, 6, and 8.

The study product was a heparinoid moisturizer containing glycerin, petrolatum, and squalene as the major additives (Hirudoid Lotion 0.3%; Maruho Co., Ltd., Osaka, Japan). The study product was applied to dry and itchy areas, including the hypochondriac region (the site at which the WCSC was measured), in principle twice daily in the morning and after taking a bath (or at bedtime on a day without bathing), but once daily after dialysis on the days of assessments, according to the study schedule in Figure 1. The amount to be applied was based on the fingertip unit,^14^ with 0.5 g regarded as the amount required to cover the area of two palms. This was originally the standard amount of topical steroid application, and its applicability for moisturizers has been reported previously.^15^ Video‐assisted education was provided to all participants to demonstrate appropriate application methods. Any oral, injectable, or topical medication, including antipruritic medications, that was being used at the start of study treatment (week 0) and required continuation during the study period was continued with no change to the dosage regimen. Additionally, throughout the study period, no change was allowed in the dialysis conditions (type of dialysis, frequency per week), and concomitant use of any other heparinoid‐containing products, urea preparations, or petrolatum was prohibited.

### 2.4 Study population

The study participants were clinically stable patients with chronic kidney disease undergoing hemodialysis. Their age ranged from 20 to 80 years on the day on which they gave informed consent, and all had been diagnosed with xerosis in the hypochondriac region and had dialysis‐associated pruritus at the start of the study treatment. Fully informed written consent was obtained from all participants before enrollment. Patients were excluded if they had used any moisturizer (heparinoid‐containing products, urea preparations, or petrolatum) within 3 weeks before the scheduled start day of the study treatment, had a history of allergy to any heparinoid‐containing product, or were otherwise ineligible for this study in the opinion of the investigator. The target sample size was set to 38 participants per group based on the results of our preceding exploratory study,^13^ assuming an intergroup difference of 7.61 in the mean WCSC at week 4 (the primary endpoint of this study) with a standard deviation of 9 for both groups, a 1:1 randomization ratio, a 5% two‐sided significance level, 90% power, and allowance for a 20% dropout rate.

### 2.5 Study variables

#### 2.5.1 Patient demographics

Sex, age, height, and body weight were recorded on the day on which participants gave informed consent.

#### 2.5.2 Adherence to study treatment

Adherence to study treatment was assessed at weeks 1, 2, 3, 4, 6, and 8 based on the patient's self‐completed diary and an interview with the participant. The following five categories were used to assess adherence: “completely or nearly as directed (applied on ≥90% of occasions),” “fairly as directed (applied on 75% to <90% of occasions),” “at least half as directed (applied on 50% to <75% of occasions),” “less than half as directed (applied on <50% of occasions),” and “never applied.”

#### 2.5.3 Efficacy outcomes

##### Primary endpoint: WCSC at week 4

The WCSC was measured on each day of evaluation 1 to 2 hours after the start of a dialysis session. One site in either the left or right hypochondriac region was designated as the measurement site for each participant. The measurement was conducted using the same instrument as in the previous study^13^ (Corneometer and Multi Display Devices MDD4; Courage + Khazaka Electronic GmbH, Köln, Germany). Measurement of the WCSC by the corneometer is based on the electrical capacitance of the skin surface (at approximately 15 µm depth) and values ranging from 0 to 120 arbitrary units (AU) were delivered. On each day of evaluation, the WCSC was measured five times and the average of the five measurements was recorded.

##### Secondary endpoint: Skin dryness score

A board‐certified dermatologist centrally rated the severity of skin dryness on a five‐point scale according to the criteria shown in Supporting Information Table S1, based on photographs of the hypochondriac region (at the measurement site for WCSC) taken on each day of evaluation.

##### Secondary endpoint: Pruritus visual analog scale score

Participants rated skin pruritus at the study product application site on a 100‐mm visual analog scale (VAS; 0 mm indicating no pruritus and 100 mm indicating the worst possible pruritus) before the start of dialysis on each day of evaluation.

##### Secondary endpoint: DLQI score

The participants assessed skin disease‐specific QOL at weeks 0, 2, 4, and 8 by completing the DLQI questionnaire,^5^ which comprises six domains: “Symptoms and feelings” (questions 1 and 2), “Daily activities” (questions 3 and 4), “Leisure activities” (questions 5 and 6), “Work and school” (question 7), “Personal relationships” (questions 8 and 9), and “Treatment” (question 10).

##### Safety outcomes

Adverse events that occurred during weeks 0–8 were investigated.

### 2.6 Statistical analysis

The efficacy analysis set comprised participants who used the study product during weeks 0 to 8 and for whom any efficacy data were available. The safety analysis set comprised participants for whom any safety data were available after using the study product.

The analysis of patient demographics included all patients enrolled in the study, with calculation of the summary statistics by group. The primary efficacy endpoint was analyzed with calculation of summary statistics by group, and intergroup comparison was performed with the unpaired *t*‐test. The secondary efficacy endpoints were summarized for each day of evaluation, with calculation of summary statistics by group. The skin dryness score and the pruritus VAS score were compared between the groups using Wilcoxon's rank‐sum test or within groups using Wilcoxon's signed‐rank test (period I [weeks 1 and 2] vs week 0, and period II [weeks 3, 4, 6, and 8] vs week 2). Other variables were compared between the groups by the unpaired *t*‐test or within a group by the paired *t*‐test (period I vs week 0 and period II vs week 2). In the intergroup comparison, the estimated difference and its 95% confidence interval were calculated. A two‐sided test was used for all statistical analysis. The significance level was set at 5% for intergroup comparisons and 2.5% or 1.25% for intragroup comparisons, adjusted for multiplicity using the Bonferroni method. In the safety evaluation, the number of participants who had adverse events, the numbers of adverse events, and the incidence of adverse events in periods I and II were calculated by group and overall.

## 3 RESULTS

### 3.1 Patient demographics

**FIGURE 2:**
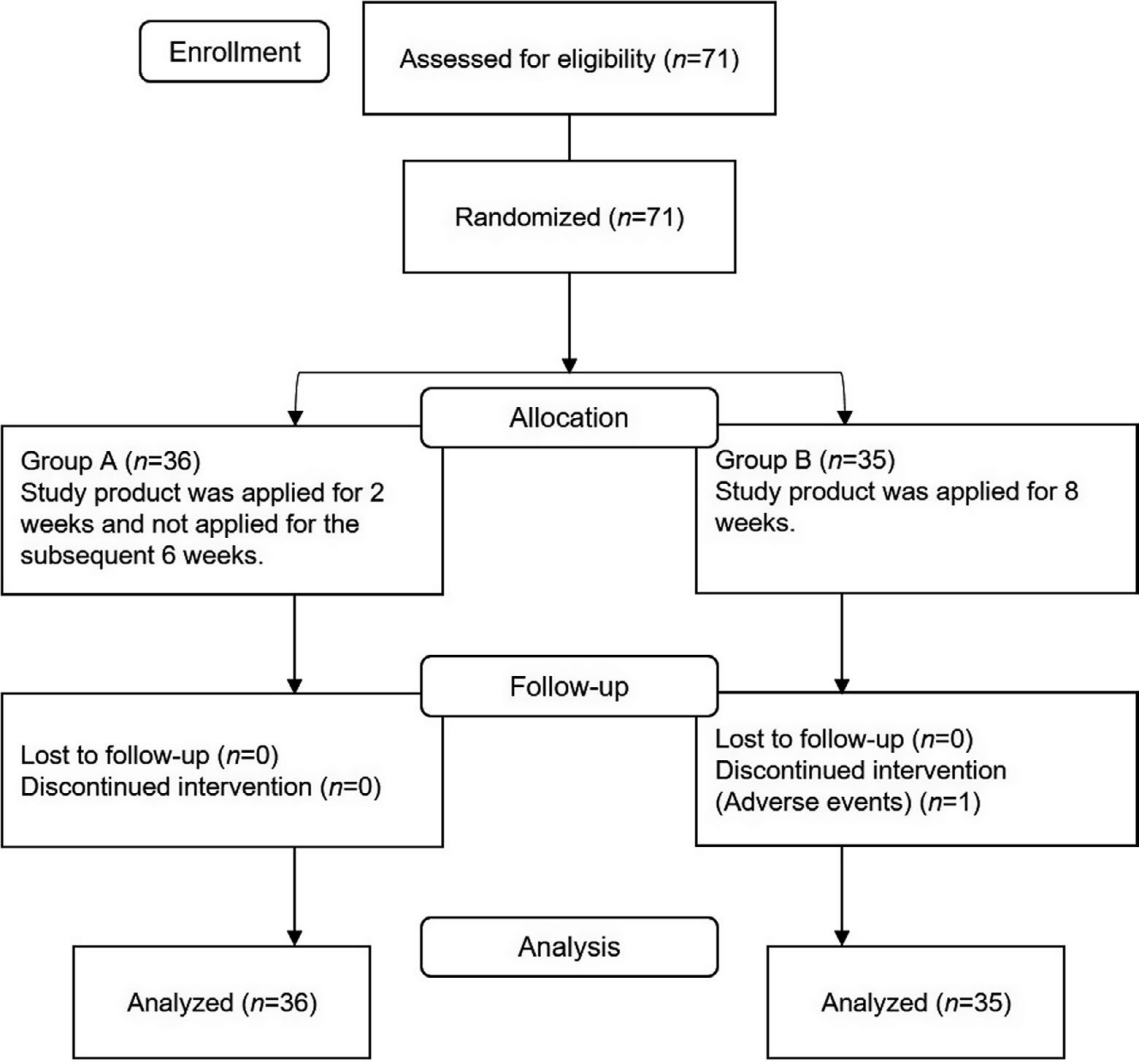
Disposition of patients. Patient disposition is shown using a CONSORT 2010 flow diagram

Although the target sample size was 76, enrollment was stopped after 71 patients were enrolled because almost no participants dropped out and the required number for analysis was reached early. Of the 71 enrolled patients, one was withdrawn from the study because of adverse events and the remaining 70 participants completed the study (Figure 2). All enrolled participants were included in the efficacy and safety analysis sets. Patient demographics are shown in Table 1. In both groups, men were predominant (26 men and 10 women in group A, 22 men and 13 women in group B). The mean age was 64.1 ± 10.2 years in group A and 61.6 ± 12.6 years in group B. Ten (27.8%) of 36 patients in group A and seven (20.0%) of 35 patients in group B used drugs that may affect pruritus (topical steroids, oral antihistamines, and oral nalfurafine hydrochloride) at the start of the study treatment.

**TABLE 1:** Patient demographics

	Group A (n = 36)	Group B (n = 35)	Total (n = 71)
Sex			
Male	26 (72.2)	22 (62.9)	48 (67.6)
Female	10 (27.8)	13 (37.1)	23 (32.4)
Age, years	64.1 ± 10.2	61.6 ± 12.6	62.9 ± 11.4
Height, cm^a^	161.9 ± 9.5	162.0 ± 7.2	161.9 ± 8.3
Weight, kg	64.9 ± 15.4	60.7 ± 15.9	62.8 ± 15.7
Antipruritic medication^b^	10 (27.8)	7 (20.0)	17 (23.9)

Note

Data are presented as n (%) or mean ± standard deviation.

^a^ Group A, n = 29; group B, n = 30.

^b^ Number (percentage) of patients receiving topical steroids, oral antihistamines, or oral nalfurafine hydrochloride at the start of study treatment.

### 3.2 Adherence to study treatment

Adherence to study treatment is summarized in Table 2. During the treatment period (period I for group A, periods I and II for group B) the study product was applied “Completely or nearly as prescribed” in 77.8%–91.4% of participants. Outside the treatment period (period II for group A), the study product was “Never applied” in 94.4%–100.0% of participants.

**TABLE 2:** Adherence to study treatment

	Period I			Period II		
	Week 1	Week 2	Week 3	Week 4	Week 6	Week 8
Group A	n = 36	n = 36	n = 36	n = 36	n = 36	n = 36
Completely or nearly as directed^a^	29 (80.6)	28 (77.8)	0 (0.0)	0 (0.0)	0 (0.0)	0 (0.0)
Fairly as directed^b^	5 (13.9)	6 (16.7)	0 (0.0)	0 (0.0)	0 (0.0)	0 (0.0)
At least half as directed^c^	2 (5.6)	2 (5.6)	0 (0.0)	0 (0.0)	0 (0.0)	0 (0.0)
Less than half as directed^d^	0 (0.0)	0 (0.0)	2 (5.6)	1 (2.8)	1 (2.8)	0 (0.0)
Never applied	0 (0.0)	0 (0.0)	34 (94.4)	35 (97.2)	35 (97.2)	36 (100.0)
Group B	n = 35	n = 35	n = 35	n = 34	n = 34	n = 34
Completely or nearly as directed^a^	32 (91.4)	32 (91.4)	28 (80.0)	28 (82.4)	27 (79.4)	28 (82.4)
Fairly as directed^b^	1 (2.9)	2 (5.7)	4 (11.4)	5 (14.7)	5 (14.7)	3 (8.8)
At least half as directed^c^	2 (5.7)	1 (2.9)	2 (5.7)	1 (2.9)	2 (5.9)	3 (8.8)
Less than half as directed^d^	0 (0.0)	0 (0.0)	0 (0.0)	0 (0.0)	0 (0.0)	0 (0.0)
Never applied	0 (0.0)	0 (0.0)	0 (0.0)	0 (0.0)	0 (0.0)	0 (0.0)

Note

Data are presented as n (%). In group A, the study product was to be applied during period I and not during period II. In group B, the study product was to be applied in both periods I and II. Video‐assisted education regarding the amount and methods of study product application was provided before the start of the treatment period.

^a^ Applied on ≥90% of occasions.

^b^ Applied on 75% to <90% of occasions.

^c^ Applied on 50% to <75% of occasions.

^d^ Applied on <50% of occasions.

### 3.3 WCSC

**FIGURE 3:**
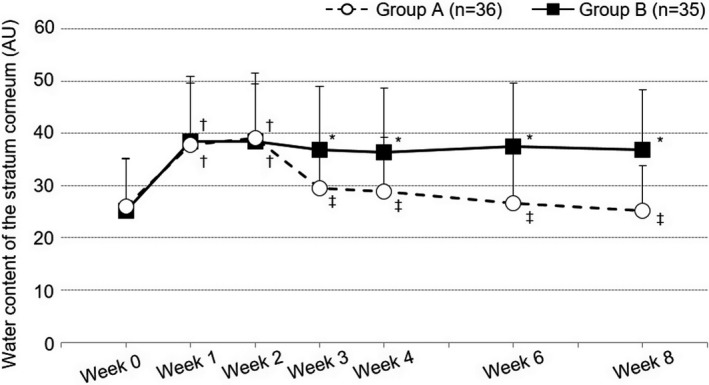
Water content of the stratum corneum. Open circles and filled squares indicate mean values of the water content in the stratum corneum, and horizontal bars indicate the standard deviation. †*P* < 0.025 vs week 0, ‡*P* < 0.0125 vs week 2 (paired *t*‐test, significance level 2.5% in period I and 1.25% in period II). *<0.05 vs group A (unpaired *t*‐test, significance level 5%). AU, arbitrary unit

The WCSC over time is shown in Figure 3 and Table S2. The mean WCSC at week 4 (primary endpoint) was 28.8 ± 10.4 AU in group A and 36.4 ± 12.2 AU in group B (*P* = 0.0068, estimated difference −7.6, 95% confidence interval −13.0 to −2.17). Compared with the value at week 0 (26.0 ± 9.0 AU in group A, 25.2 ± 10.0 AU in group B), the WCSC was significantly increased by the end of period I (week 2) in both groups (39.0 ± 12.5 AU in group A, 38.5 ± 11.0 AU in group B; *P* < 0.00001 for both vs week 0). After week 2, the value in group B was maintained until the end of period II (week 8) (36.8 ± 11.5 AU; *P* = 0.9532 vs week 2), while the value in group A began decreasing and finally reached 25.1 ± 8.6 AU at week 8 (*P* < 0.00001 vs week 2). The room temperature and relative humidity (RH) at the start of the measurement of WCSC were 18.2–28.5°C and 18.7–61.3%RH, respectively.

### 3.4 Skin dryness score

**FIGURE 4:**
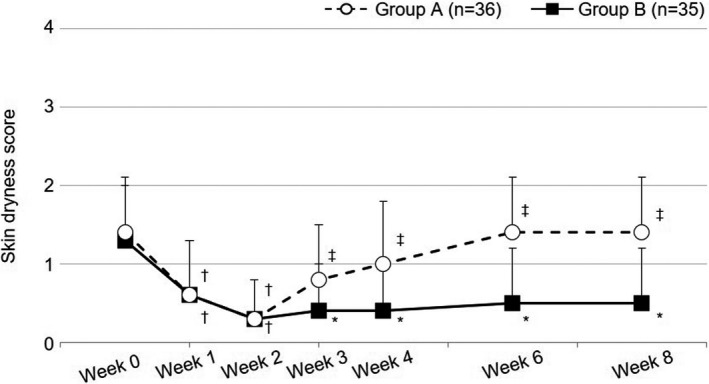
Skin dryness score. Open circles and filled squares indicate mean values of the skin dryness score, and horizontal bars indicate the standard deviation. †*P *< 0.025 vs week 0, ‡*P *< 0.0125 vs week 2 (Wilcoxon's signed‐rank test, significance level 2.5% in period I and 1.25% in period II). *<0.05 vs group A (Wilcoxon's rank‐sum test, significance level 5%)

The skin dryness score over time is shown in Figure 4 and Table S3. At week 2, the score significantly decreased in both groups (group A: 1.4 ± 0.7 at week 0 and 0.3 ± 0.5 at WEEK 2, *P* < 0.00001; group B: 1.3 ± 0.7 at week 0 and 0.3 ± 0.5 at week 2, *P* < 0.00001). After week 2, the score showed a tendency to increase only in group A.

### 3.5 Pruritus VAS score

**FIGURE 5:**
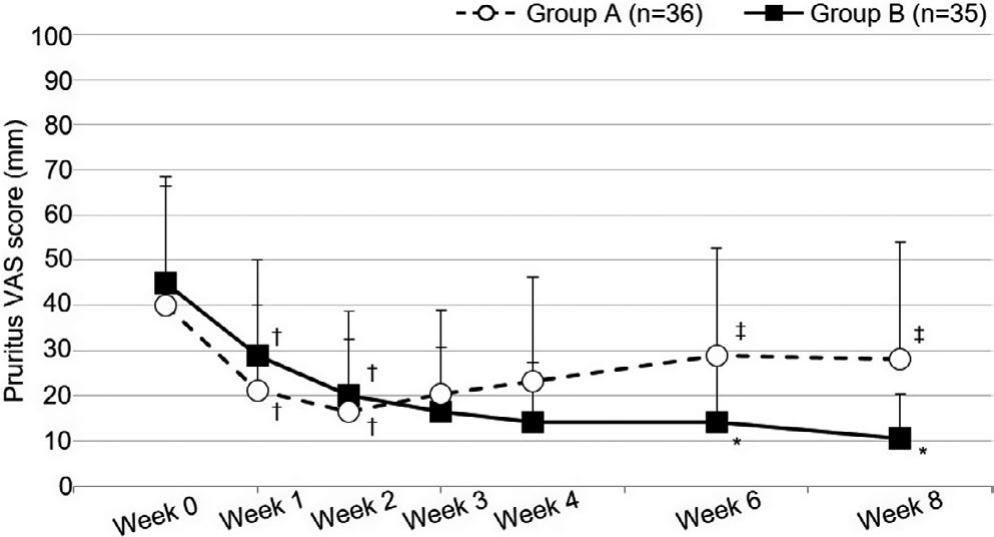
Pruritus VAS score. Open circles and filled squares indicate mean values of the pruritus VAS score, and horizontal bars indicate the standard deviation. †*P* < 0.025 vs week 0, ‡*P* < 0.0125 vs week 2 (Wilcoxon's signed‐rank test, significance level 2.5% in period I and 1.25% in period II). *<0.05 vs group A (Wilcoxon's rank‐sum test; significance level 5%). VAS, visual analog scale

The pruritus VAS score over time is shown in Figure 5 and Table S4. In both groups, the pruritus VAS score significantly decreased from week 0 (40.0 ± 26.3 mm in group A, 44.8 ± 23.7 mm in group B) to the end of period I (week 2) (16.5 ± 16.1 mm in group A, 20.1 ± 18.7 mm in group B; *P* < 0.00001 for both vs week 0). After week 2, the score continued to decrease in group B, but began increasing in group A. At the end of period II (week 8), the value was significantly lower in group B (10.5 ± 9.9 mm) than in group A (28.0 ± 26.0 mm) (*P* = 0.0018, estimated difference 17.5, 95% confidence interval 8.02–27.0).

### 3.6 DLQI score

**FIGURE 6:**
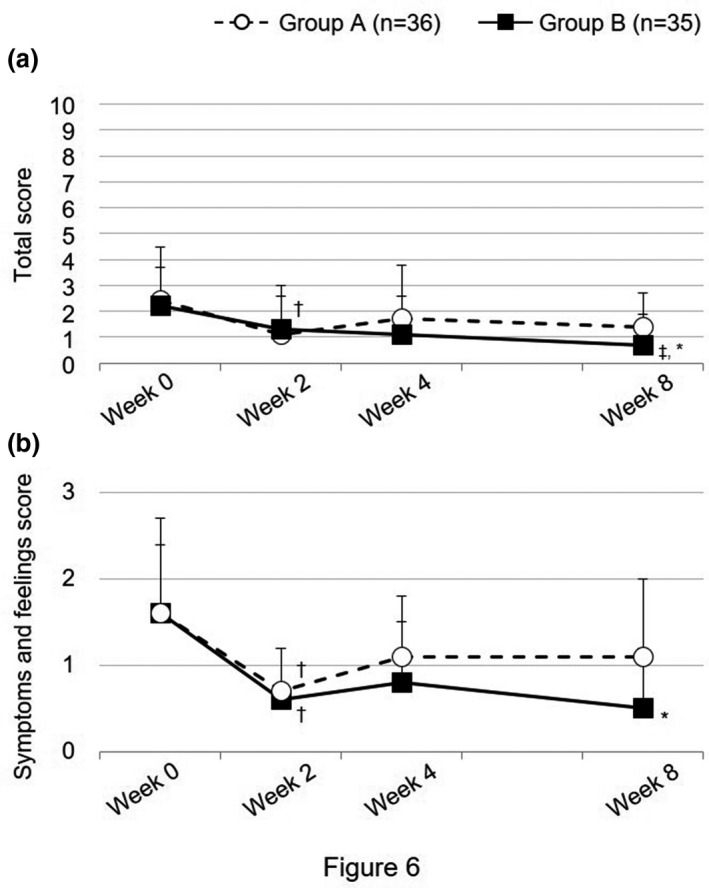
Dermatology life quality index (DLQI) score. Open circles and filled squares indicate mean values of the DLQI score, and horizontal bars indicate the standard deviation. (a) Total score. (b) Symptoms and feelings subscore. †*P* < 0.05 vs week 0, ‡*P* < 0.025 vs week 2 (paired *t*‐test, significance level 5% in period I and 2.5% in period II). *<0.05 vs group A (unpaired *t*‐test, significance level 5%)

The DLQI score over time is shown in Figure 6 and Table S5. At week 8, the total DLQI score was significantly lower at 0.7 ± 1.2 in group B compared with 1.4 ± 1.3 in group A (*P* = 0.0278, estimated difference 0.67, 95% confidence interval 0.07‐1.26). By domain, the “Symptoms and feelings” subscore over time was similar to that of the total score, with a significant intergroup difference at week 8 (1.1 ± 0.9 in group A, 0.5 ± 0.7 in group B; *P* = 0.0089, estimated difference 0.53, 95% confidence interval 0.14‐0.92). For the other domains (Daily activities, Leisure, Work and school, Personal relationships, and Treatment), the score remained <1 throughout the study period and was essentially unchanged (data not shown).

### 3.7 Adverse events

In total, 126 adverse events were reported in 51 of the 71 participants in the safety analysis set (Table 3). One participant in group B had two adverse events (rash and erythema) that might have been related to the study product, and this patient was withdrawn from the study. These adverse events resolved after discontinuation of the study product. In total, six serious adverse events were reported in four participants; none of these adverse events were related to the study product.

**TABLE 3:** List of adverse events

	Group A (n = 36)		Group B (n = 35)	
	n (%)	Number of events	n (%)	Number of events
Infections and infestations	9 (25.0)	13	9 (25.7)	10
Blood and lymphatic system disorders	0 (0.0)	0	2 (5.7)	2
Immune system disorders	1 (2.8)	1	0 (0.0)	0
Endocrine disorders	1 (2.8)	1	1 (2.9)	1
Metabolism and nutrition disorders	1 (2.8)	1	3 (8.6)	3
Psychiatric disorders	1 (2.8)	1	1 (2.9)	1
Nervous system disorders	2 (5.6)	2	1 (2.9)	1
Eye disorders	2 (5.6)	2	4 (11.4)	4
Cardiac disorders	4 (11.1)	5	1 (2.9)	1
Vascular disorders	4 (11.1)	4	1 (2.9)	1
Respiratory, thoracic, and mediastinal disorders	7 (19.4)	12	1 (2.9)	4
Gastrointestinal disorders	4 (11.1)	4	8 (22.9)	10
Skin and subcutaneous tissue disorders	3 (8.3)	5	4 (11.4)	6
Musculoskeletal and connective tissue disorders	4 (11.1)	4	2 (5.7)	2
General disorders and administration site conditions	1 (2.8)	1	2 (5.7)	2
Investigations	3 (8.3)	8	5 (14.3)	5
Injury, poisoning, and procedural complications	4 (11.1)	4	2 (5.7)	5
Total	26 (72.2)	68	25 (71.4)	58

Note

Adverse events summarized by system organ class (MedDRA Version 21.1).

## 4 DISCUSSION

Heparinoid‐containing products are widely used for xerosis caused by various factors, including dialysis, in Japan. However, no reports other than our previous study^13^ have described the effectiveness of such products for xerosis in patients undergoing dialysis as evaluated with an objective indicator such as the WCSC. In the present study, we investigated the time course of the severity of skin dryness and associated changes in pruritus and QOL with comparison between two groups undergoing different lengths of treatment and with an adequate sample size set based on preceding exploratory study results on the WCSC at week 4.^13^

In this study, xerosis measured by WCSC and skin dryness score improved from week 1 of application of the heparinoid‐containing product and worsened from 1 week after discontinuation of the application. Additionally, between the two groups of patients who continued and discontinued application of the heparinoid‐containing product, the WCSC at week 4 (primary endpoint) and all other time points of evaluation after week 3 showed significant differences. These findings suggest that continued use of a heparinoid‐containing product can improve xerosis in patients undergoing dialysis and maintain the improvement. Moreover, pruritus and xerosis synchronously improved or worsened, which indicates that xerosis plays a role in pruritus in patients undergoing dialysis as previously reported and that improvement of xerosis with a heparinoid‐containing product can lead to reduced pruritus. The DLQI analysis results suggested that treatment of xerosis in patients undergoing dialysis can lead not only to reduced pruritus but also better QOL. In particular, the time course was similar between the “Symptoms and feelings” subscore and the DLQI total score, which indicates that xerosis and pruritus in patients undergoing dialysis may affect the psychological health of the patients. During the study period, one participant experienced two adverse reactions, and both were known reactions to the study product, thus raising no new safety issues with the use of this heparinoid‐containing product for xerosis in patients undergoing dialysis.

In this study, all efficacy endpoints showed clear differences in the skin condition over time between participants who continued application of the heparinoid‐containing product and those who discontinued the application, partly because of the favorable treatment adherence in >75% of the participants. In particular, the patients received pretreatment education regarding the appropriate application methods based on the fingertip unit ^14^ every day throughout the study period. This probably helped to clarify the appropriate amount of the heparinoid‐containing product to be applied, thereby leading to the observed adequate effectiveness in the treatment period. The results thus imply the importance of proper moisturizer use for maintaining good skin condition in patients undergoing dialysis.

The strengths of this study include its design, which was based on the results of an exploratory study. Additionally, the effect of the skin treatment on dialysis patients’ QOL was evaluated using DLQI and the patients’ skin symptoms were visually evaluated by a dermatologist. The limitations of the study included a lack of comparison with a placebo, the use of moisturizers other than heparinoid‐containing products for study treatment, and a lack of enrollment of patients undergoing peritoneal dialysis. Additionally, the efficacy of heparinoid moisturizer in patients with senile xerosis was reported by Hayama et al.^16^ Given that most of the subjects were elderly, the results of this study may reflect an improvement in xerosis accompanying not only dialysis, but also aging. Moreover, xerosis can be caused by environmental factors.^17^ Further research on the characteristics of the study subjects and the study season is therefore required.

This study demonstrated that proper application of a heparinoid‐containing product in patients undergoing dialysis can improve xerosis and reduce associated pruritus, and that continued application is required to maintain the improved skin condition. Additionally, improvement in the symptoms of xerosis and pruritus was shown to lead to improved QOL of the participants. It is therefore important to not only prescribe moisturizers to dialysis patients with xerosis, but also to support them in terms of medication compliance.

## CONFLICTS OF INTEREST

The Kidney Disease Clinic of Nippon Medical School, Department of Nephrology of Nippon Medical School, Department of Dermatology of Nippon Medical School, Koyama Memorial Hospital, and Moka Hospital were paid by Maruho Co., Ltd for conducting this study. Yukie Yoshida, who presented the study results at the American Society of Nephrology (ASN) Kidney Week 2019, was paid by Maruho Co., Ltd for travel and accommodation expenses and remuneration. Shuichi Tsuruoka was paid by Maruho Co., Ltd for his advisory tasks in this study. Momoyo Kishida, Hiroshi Nakamura, and Akira Kanakubo are employees of Maruho Co., Ltd. The other authors have no conflicts of interest to declare.

## PRIOR PRESENTATION

Parts of the results of this study were presented at the ASN Kidney Week 2019 (November 2019, Washington, DC) and the 40th Annual Scientific Meeting of the Japanese Society of Clinical Pharmacology and Therapeutics (December 2019, Tokyo).

## Supporting information

Table S1

Table S2

Table S3

Table S4

Table S5
