# Supplementary material for: Efficacy of a moisturizer for pruritus accompanied by xerosis in patients undergoing dialysis: A multicenter, open‐label, randomized verification study
Source: J Dermatol. 2021 May 26;48(9):1327–35. doi: 10.1111/1346-8138.15950 (PMC8453556; doi:10.1111/1346-8138.15950)
Supplement: Supplementary file 1 — Table S1 [file JDE-48-1327-s004.pdf]

Supplementary Table 1 Skin dryness score-rating criteria

Severity score	Rating criteria
0 (None)	No dryness observed
1 (Very mild)	Very mild dryness observed
2 (Mild)	Mild dryness observed
3 (Moderate)	Distinct dryness observed
4 (Severe)	Severe dryness observed

A board-certified dermatologist evaluated the severity of skin dryness according to the criteria shown in the table, based on photographs taken on the evaluation days.
