# Supplementary material for: Efficacy of a moisturizer for pruritus accompanied by xerosis in patients undergoing dialysis: A multicenter, open‐label, randomized verification study
Source: J Dermatol. 2021 May 26;48(9):1327–35. doi: 10.1111/1346-8138.15950 (PMC8453556; doi:10.1111/1346-8138.15950)
Supplement: Supplementary file 2 — Table S2 [file JDE-48-1327-s003.pdf]

Supplementary Table 2 Water content of the stratum corneum

		Group A (n=36)	Group B (n=35)	Inter-group comparison P-value
Period I	Week 0	26.0 ± 9.0	25.2 ± 10.0	N/A
	Week 1	37.8 ± 11.8<br>P<0.0001	38.4 ± 12.5[a]<br>P<0.0001	0.8362
	Week 2	39.0 ± 12.5<br>P<0.0001	38.5 ± 11.0<br>P<0.0001	0.8391
Period II	Week 3	29.4 ± 8.5<br>P<0.0001	36.8 ± 12.2[a]<br>P=0.5846	0.0050
	Week 4	28.8 ± 10.4<br>P<0.0001	36.4 ± 12.2[b]<br>P=0.5203	0.0068
	Week 6	26.6 ± 9.5<br>P<0.0001	30.9 ± 15.5[a]<br>P=0.9080	<0.0001
	Week 8	25.1 ± 8.6<br>P<0.0001	36.8 ± 11.5[a]<br>P=0.9532	<0.0001

Mean ± standard deviation presented for measured value of water content of the stratum corneum. For intra-group comparison, P-values vs. baseline (Week 0 for Period I, Week 2 for Period II) (by paired t-test) are shown. For inter-group comparison, P-values between two groups at respective time points (by unpaired t-test) are shown.

[a]n=34, [b]n=33.

N/A, not applicable.
