# Supplementary material for: Efficacy of a moisturizer for pruritus accompanied by xerosis in patients undergoing dialysis: A multicenter, open‐label, randomized verification study
Source: J Dermatol. 2021 May 26;48(9):1327–35. doi: 10.1111/1346-8138.15950 (PMC8453556; doi:10.1111/1346-8138.15950)
Supplement: Supplementary file 3 — Table S3 [file JDE-48-1327-s005.pdf]

Supplementary Table 3 Skin dryness score

		Group A (n=36)	Group B (n=35)	Inter-group comparison P-value
Period I	Week 0	1.4 ± 0.7	1.3 ± 0.7	N/A
	Week 1	0.6 ± 0.7<br>P<0.0001	0.6 ± 0.7[a]<br>P<0.0001	0.9267
	Week 2	0.3 ± 0.5<br>P<0.0001	0.3 ± 0.5<br>P<0.0001	0.5609
Period II	Week 3	0.8 ± 0.7<br>P<0.0001	0.4 ± 0.6[a]<br>P=1.0000	0.0292
	Week 4	1.0 ± 0.8<br>P<0.0001	0.4 ± 0.6[b]<br>P=1.0000	0.0004
	Week 6	1.4 ± 0.7<br>P<0.0001	0.5 ± 0.7[b]<br>P=0.3125	<0.0001
	Week 8	1.4 ± 0.7<br>P<0.0001	0.5 ± 0.7[a]<br>P=0.2344	<0.0001

Mean ± standard deviation shown for skin dryness score. For intra-group comparison, P-values vs. baseline (Week 0 for Period I, Week 2 for Period II) (by Wilcoxon's signed-rank test) are shown. For inter-group comparison, P-values between two groups at respective time points (by Wilcoxon's rank-sum test) are shown.
[a]n=34, [b]n=33.
N/A, not applicable.
