# Supplementary material for: Efficacy of a moisturizer for pruritus accompanied by xerosis in patients undergoing dialysis: A multicenter, open‐label, randomized verification study
Source: J Dermatol. 2021 May 26;48(9):1327–35. doi: 10.1111/1346-8138.15950 (PMC8453556; doi:10.1111/1346-8138.15950)
Supplement: Supplementary file 4 — Table S4 [file JDE-48-1327-s002.pdf]

**Supplementary Table 4** Pruritus VAS score

| | | Group A ($n$=36) | Group B ($n$=35) | Inter-group comparison $P$-value |
|---|---|---|---|---|
| Period I | Week 0 | 40.0 ± 26.3 | 44.8 ± 23.7 | N/A |
| | Week 1 | 21.1 ± 19.0<br>$P$<0.0001 | 28.8 ± 21.4[b]<br>$P$=0.0005 | 0.1583 |
| | Week 2 | 16.5 ± 16.1<br>$P$<0.0001 | 20.1 ± 18.7<br>$P$<0.0001 | 0.2591 |
| Period II | Week 3 | 20.4 ± 18.5[a]<br>$P$=0.1353 | 16.4 ± 14.2[b]<br>$P$=0.3179 | 0.4350 |
| | Week 4 | 23.3 ± 22.8[a]<br>$P$=0.1122 | 14.0 ± 13.3[c]<br>$P$=0.0423 | 0.0790 |
| | Week 6 | 28.9 ± 23.8[a]<br>$P$=0.0022 | 14.2 ± 16.6[b]<br>$P$=0.1010 | 0.0037 |
| | Week 8 | 28.0 ± 26.0[a]<br>$P$=0.0109 | 10.5 ± 9.9[b]<br>$P$=0.0458 | 0.0018 |

Mean ± standard deviation shown for pruritus VAS score. For intra-group comparison, $P$-values vs. baseline (Week 0 for Period I, Week 2 for Period II) (by Wilcoxon's signed-rank test) are shown. For inter-group comparison, $P$-values between two groups at respective time points (by Wilcoxon's rank-sum test) are shown.
[a]$n$=35, [b]$n$=34, [c]$n$=33.
VAS, visual analog scale; N/A, not applicable.
