# Supplementary material for: Efficacy of a moisturizer for pruritus accompanied by xerosis in patients undergoing dialysis: A multicenter, open‐label, randomized verification study
Source: J Dermatol. 2021 May 26;48(9):1327–35. doi: 10.1111/1346-8138.15950 (PMC8453556; doi:10.1111/1346-8138.15950)
Supplement: Supplementary file 5 — Table S5 [file JDE-48-1327-s001.pdf]

Supplementary Table 5 DLQI score

		Total score			Symptoms and Feelings subscore		
		Group A (*n*=36)	Group B (*n*=35)	Inter-group comparison *P*-value	Group A (*n*=36)	Group B (*n*=35)	Inter-group comparison *P*-value
Period I	Week 0	2.4 ± 2.1	2.2 ± 1.5	N/A	1.6 ± 1.1	1.6 ± 0.8	N/A
	Week 2	1.1 ± 1.5 *P*<0.001	1.3 ± 1.7 *P*=0.1919	0.5470	0.7 ± 0.5 *P*<0.0001	0.6 ± 0.6 *P*<0.0001	0.3642
Period II	Week 4	1.7 ± 2.1[a] *P*=0.0400	1.1 ± 1.5[b] *P*=0.0332	0.1654	1.1 ± 0.7[a] *P*=0.0557	0.8 ± 0.7[b] *P*=0.7679	0.0594
	Week 8	1.4 ± 1.3[a] *P*=0.4145	0.7 ± 1.2[c] *P*=0.0003	0.0278	1.1 ± 0.9[a] *P*=0.2951	0.5 ± 0.7[c] *P*=0.4126	0.0089

Mean ± standard deviation shown for the DLQI total score and the "Symptoms and feelings" subscore. For intra-group comparison, *P*-values vs. baseline (Week 0 for Period I, Week 2 for Period II) (by paired *t*-test) are shown. For inter-group comparison, *P*-values between two groups at respective time points (by unpaired *t*-test) are shown.
[a]*n*=35, [b]*n*=33, [c]*n*=34.

DLQI, Dermatology Life Quality Index; N/A, not applicable.
